# Supplementary material for: Hard Coating Materials Based on Photo-Reactive Silsesquioxane for Flexible Application: Improvement of Flexible and Hardness Properties by High Molecular Weight
Source: Polymers (Basel). 2021 May 13;13(10):1564. doi: 10.3390/polym13101564 (PMC8153148; doi:10.3390/polym13101564)
Supplement: Supplementary file 1 [file polymers-13-01564-s001.zip › polymers-1211192-supplementary.pdf]

polymers

MDPI

Hard Coating Materials based on Photo-Reactive Silsesquioxane for Flexible Application: Improvement of Flexible and Hardness Properties by High Molecular Weight

Jong Tae Leem [1, 2], Woong Cheol Seok [1], Ji Beom Yoo [2], Sangkug Lee [1] and Ho Jun Song [1,*]

[1] Green and Sustainable Materials R&D Department/Research Institute of Clean Manufacturing System, Korea Institute of Industrial Technology, 89 Yangdaegiro-gil, Ipjang-myeon, Seobuk-gu, Cheonan-si, Chungcheongnam-do, 331-822, Republic of Korea

[2] School of Advanced Materials Science and Engineering, Sungkyunkwan University, Suwon 16419, Republic of Korea

Phone : 82-41-589-8467; Fax: 82-41-589-8550; E-mail: song3026@kitech.re.kr*

*Corresponding Author

Ph. D. Ho Jun Song, E-mail : song3026@kitech.re.kr

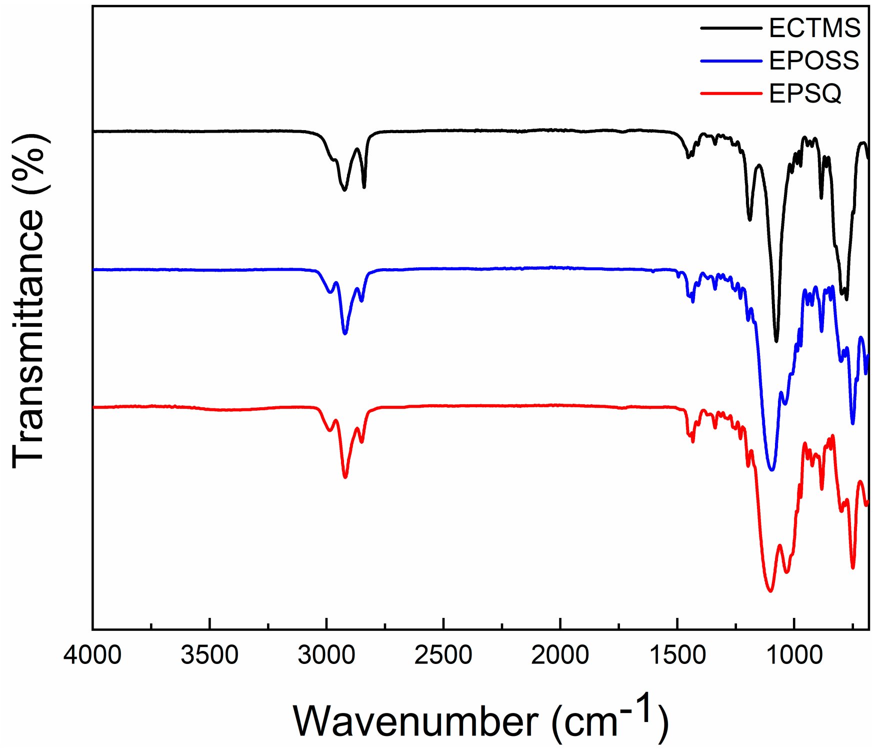

Figure S1. FT-IR spectra of ECTMS, EPOSS, and EPSQ.

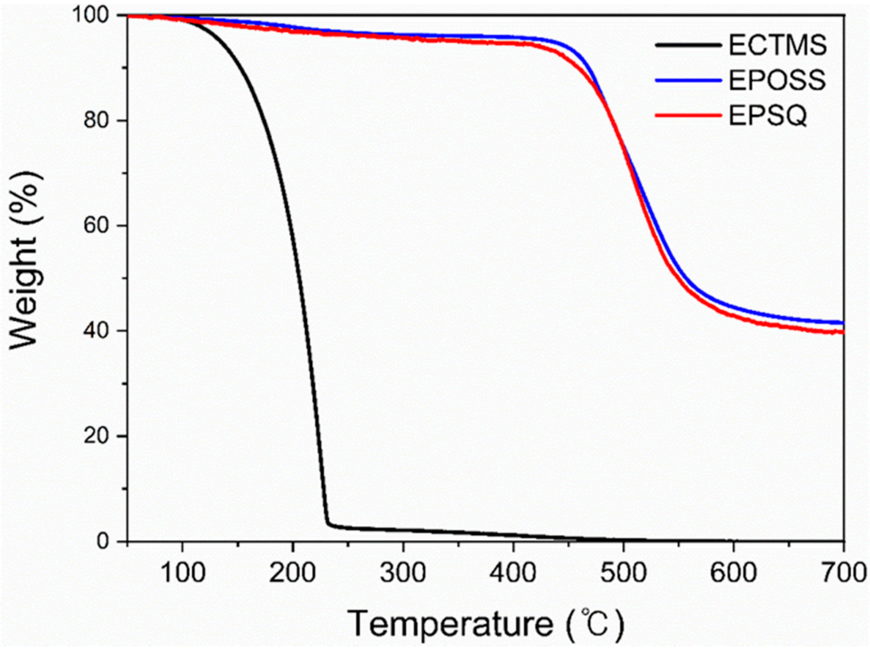

Figure S2. TGA spectra of ECTMS, EPOSS, and EPSQ.

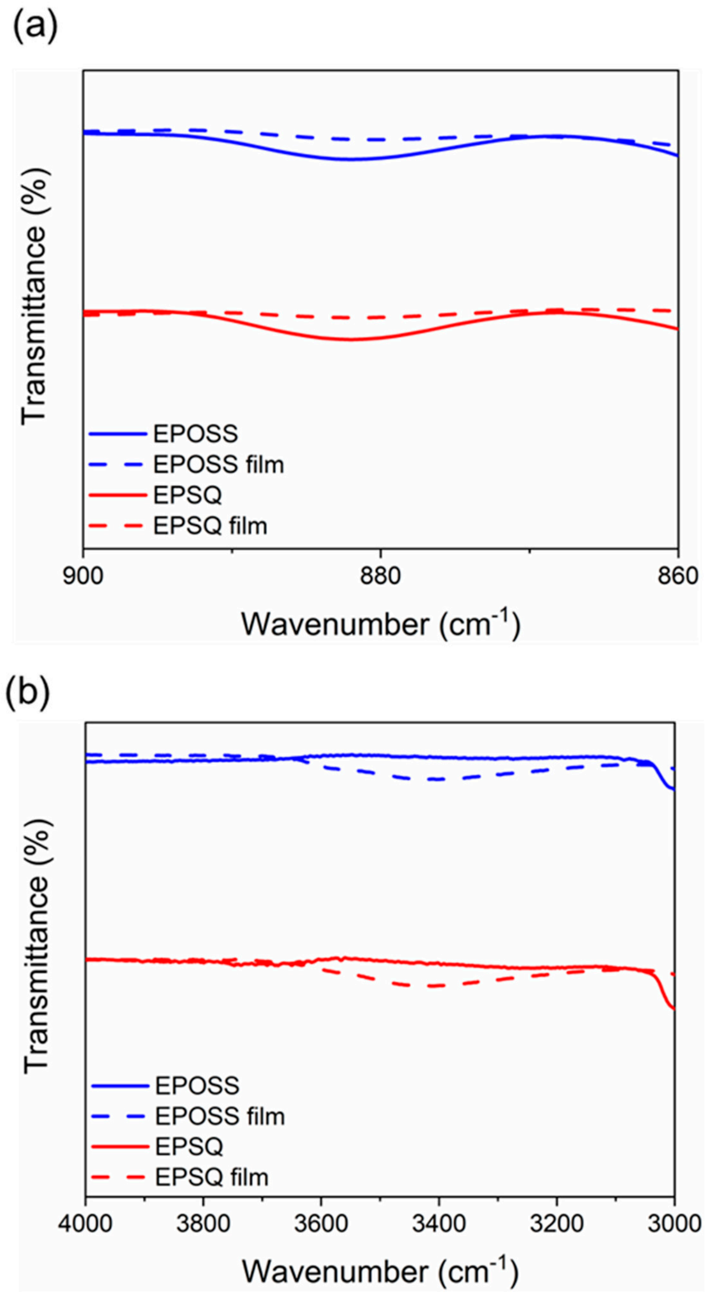

Figure S3. FT-IR spectra of UV curing for EPOSS and EPSQ: (a) epoxy group in the range of 860-900cm^{-1} and (b) hydroxyl group in the range of 3000-4000 cm^{-1}.

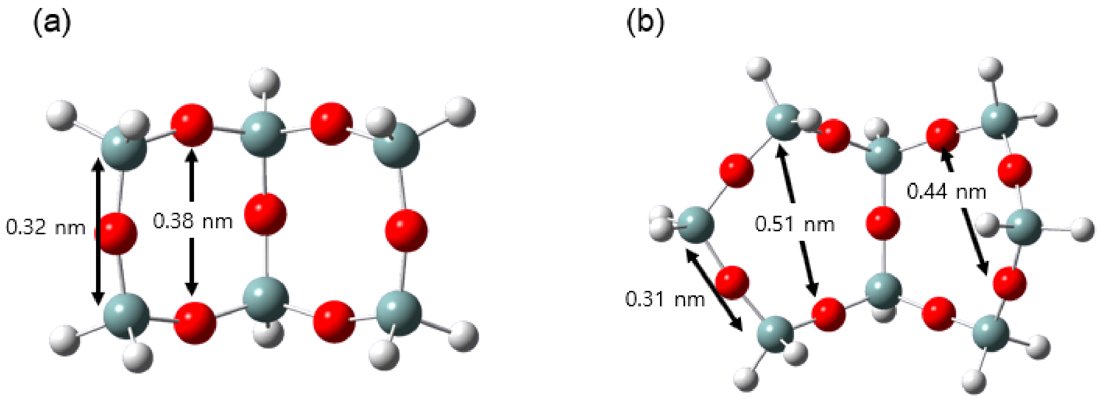

Figure S4. Molecular structures of silsesquioxane analyzed by DFT calculation (Gaussian): (a) square shape and (b) pentagon shape.

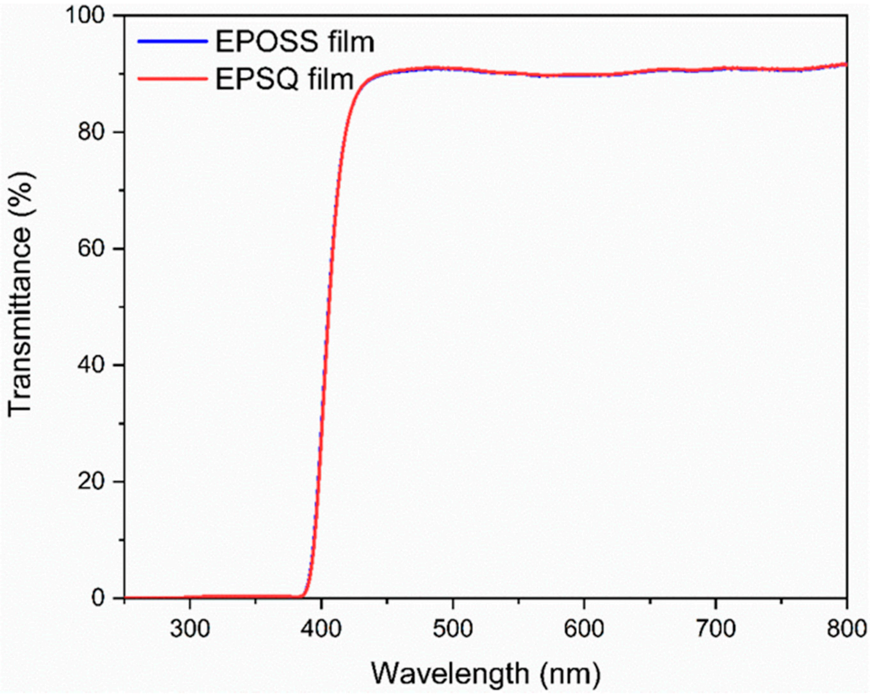

Figure S5. UV–vis spectra of EPOSS and EPSQ films.
